# Therapeutic Potential of *Conyza bonariensis* n‐Hexane Extract in Non‐Alcoholic Fatty Liver Disease: Histological, Biochemical, and In Silico Insights From a High‐Fat Diet Rat Model

**DOI:** 10.1002/fsn3.70950

**Published:** 2025-09-12

**Authors:** Bassam S. M. Al Kazman, Sadia Rana, Faiza Naseer, Taha Muhammad, Ethar Abdullah Mudhish, Mohammed A. Alshamrani, Omaish S. Alqahtani, Mater H. Mahnashi, Mohammad Saleem

**Affiliations:** ^1^ Department of Pharmacognosy College of Pharmacy, Najran University Najran Saudi Arabia; ^2^ University College of Pharmacy University of the Punjab Lahore Pakistan; ^3^ Department of Biosciences Shifa Tameer e Millat University Islamabad Pakistan; ^4^ Shalamar Medical and Dental College Lahore Pakistan; ^5^ Department of Pharmacognosy College of Pharmacy, King Khalid University Abha Kingdom of Saudi Arabia; ^6^ Department of Pharmaceutical Sciences, Pharmacy College Umm Al‐Qura University Makkah Saudi Arabia; ^7^ Department of Pharmaceutical Chemistry, College of Pharmacy King Khalid University Abha Saudi Arabia; ^8^ Yashfeen College of Pharmacy Yashfeen Education System Lahore Pakistan

**Keywords:** *Conyza bonariensis*, hepatoprotective activity, high‐fat diet, non‐alcoholic fatty liver disease

## Abstract

Non‐alcoholic fatty liver disease (NAFLD) is a rising global health concern closely linked with obesity, insulin resistance, and metabolic syndrome. Despite its prevalence, there are currently no approved pharmacological treatments. *Conyza bonariensis* (CB), a traditional medicinal plant, has demonstrated antioxidant, anti‐inflammatory, and hepatoprotective properties, making it a promising candidate for NAFLD therapy. This study investigates the therapeutic potential of CB n‐hexane extract in a rat model of high‐fat diet (HFD)‐induced NAFLD. In silico molecular docking was conducted to assess the binding affinity of major bioactive constituents to NAFLD‐associated targets, including PPAR‐α, PPAR‐γ, AMPK, and SREBP‐1c. In vivo, Sprague–Dawley rats (200–300 g) were divided into six groups: control, HFD‐induced NAFLD, Silymarin Group, and three treatment groups of three doses of 250, 500, and 750 mg/kg of CB extracts for 28 days. Biochemical markers (lipid profile, complete blood profile, hemoglobin levels, and glucose parameters) and histopathological liver changes were evaluated. In vivo, extract‐treated groups showed significant improvement in liver function tests, lipid profiles, and oxidative stress markers (*p* < 0.05), with the highest dose group (750 mg/kg) displaying histologically reduced hepatic steatosis and inflammation. CB n‐hexane extracts demonstrated significant hepatoprotective and metabolic regulatory effects in HFD‐induced NAFLD in rats, potentially mediated through modulation of PPAR and AMPK pathways. The integration of in silico and pharmacological data supports further exploration of CB as a potential natural therapeutic for NAFLD.

## Graphical abstract

This study investigates the therapeutic potential of Conyza bonariensis (CB) n‐hexane extracts in treating high‐fat diet‐induced non‐alcoholic fatty liver disease (NAFLD) in rats. Results showed significant improvements in liver function, lipid profile, and oxidative stress, particularly at the 750 mg/kg dose, supported by in‐silico docking to NAFLD‐related targets, highlighting CB as a promising natural treatment option.

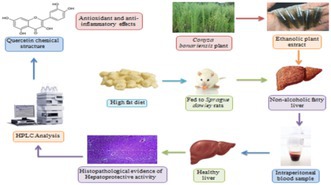

## 1 Introduction

NAFLD is the most frequent cause of liver disease and is prevalent. It is more common in males than in females, which is one of the major causes of liver failure (LaBrecque et al. 2014; Ren et al. 2023). NAFLD is a marker associated with metabolic syndrome. It is linked with chronic cardiac disease, hyperlipidemia, hypertension, and glucose intolerance (Newton et al. 2016; Blachier et al. 2013). The basic cause is unclear, but various other factors, like obesity, hyperlipidemia, diabetes, and hypertension, exist. Genetic factors associated with NAFLD lead to mutations in genes involved in protein synthesis and the export of triacylglycerol (Li et al. 2018). Environmental aspects are the leading cause of NAFLD in obese patients, including food and lifestyle, closely associated with the progression of NAFLD, affecting 70% of diabetic mellitus (DM) patients (Hashimoto et al. 2013). Females aged greater than 50 are at high risk of disease. NAFLD has no prominent symptoms in the initial stage but appears as liver failure in later stages (Noureddin et al. 2013).

The main histological characteristics for the diagnosis of NAFLD are steatosis, inflammation, and cellular ballooning (Deng et al. 2022). Ultrasound is commonly used for the diagnosis of NAFLD, whereas a CT scan is more costly compared to the ultrasound technique and has less vulnerability (Tikoo et al. 2016). MRI (Magnetic Resonance Imaging) and MRS (Magnetic Resonance Spectroscopy) are standard precision techniques in NAFLD (Chalasani et al. 2012). The patients have fatty liver upon abdominal imaging and high levels of ALT and AST (liver enzymes), which ultimately lead to liver biopsy (Byrne 2012). Symptoms are edema, inflamed blood vessels just beneath the skin, jaundice, splenomegaly, and red palms (Mehal 2013). Excessive fat accumulation causes insulin resistance, Type 2 DM, and hepatic inflammation, which in turn lead to hepatic cirrhosis and NAFLD (Jornayvaz and Shulman 2012). To alleviate the menace of NAFLD, it is necessary to treat the causes that are associated with metabolic syndrome (Perry et al. 2014). Regular daily exercise causes improvement in the symptoms of NAFLD and insulin sensitivity. NAFLD has biochemical, hematological, and oxidative stress biomarkers, which are indicators of disease onset (Watt et al. 2012). Excessively high levels of liver function tests indicate nonspecific hepatocellular damage. Hepatic steatosis occurs when there is an imbalance in fatty acid production and catabolism. Dyslipidemia and its irregular metabolism, along with chronic inflammation, would result in NAFLD and CVD (Longrigg 2013). The accumulation of triacylglycerol in the liver is the main histological and metabolic characteristic of NAFLD. Mean Platelet Volume (MPV) has a positive correlation with NAFLD; the higher the value of MPV, the greater the frequency of NASH (Valan et al. 2010). A high level of C‐reactive protein (CRP) is a protein that is synthesized mainly in the liver, as well as in adipose tissues. It is the main biomarker of NASH under abnormal physiological conditions such as obesity, metabolic syndrome, and insulin resistance (Zahoor et al. 2012). Normal function of TNF‐α causes insulin sensitivity and a low level of fatty acids in the liver (Neuman et al. 2014).

Multiple regimens are suggested for the treatment of NAFLD. Statins help in maintaining blood cholesterol levels. Thiazolidinedione has therapeutic benefits with improvement in insulin sensitivity (Saleem, Naseer, et al. 2014). Pioglitazones have benefits as they improve steatosis and lobular inflammation (Picchi et al. 2011). The rise in use of herbal and natural products has increased two to three times, mostly for primary health care, according to WHO (Farzaneh and Carvalho 2015). From early ages, herbal medicine has gained significant importance. WHO has shown that 80% of the world's population uses herbal treatment to cure various ailments. *Conyza bonariensis* (CB) is a plant belonging to the family Asteraceae, having hepatoprotective ability due to the presence of phytochemicals like polyphenols, flavonoids, steroids, quinones, coumarins, essential oils, terpenoids, anthocyanidins, saponins, alkaloids, and nitrogenous compounds (Noreen et al. 2018). It is used as an antibacterial, antidiabetic, and anthelmintic for skin infections and kidney disorders (Akhtar 2021). CB is traditionally used for cough, fever, dysentery, diarrhea, anthelmintic, astringent, eczema, ringworm, and renal disorder. It possesses certain therapeutic activities, gut modulatory activity, and anti‐inflammatory activity. On *CB*, no in vivo study of hepatoprotective activity against HFD‐induced NAFLD was performed (Saleem, Naseer, et al. 2014).

The objective of this study was to investigate the hepatoprotective potential of n‐hexane extracts of CB in the HFD‐induced non‐alcoholic fatty liver disease (NAFLD) model in rats. The study aimed to evaluate the extract's effects on liver function, lipid profile, oxidative stress, inflammatory biomarkers, and histopathological changes. Additionally, in silico molecular docking analysis was conducted to identify active phytoconstituents within the extract and their interactions with key NAFLD‐related targets, including PPAR‐α, PPAR‐γ, AMPK, and SREBP‐1c. This integrative approach was designed to provide both experimental and computational evidence for the therapeutic potential of this plant as a natural treatment strategy for NAFLD.

## 2 Materials and Methodology

Silymarin 100 mg/5 mL syrup and Multivitamins Vitrum were used. are n‐Hexane (Merck USA), Normal Saline (Medisol), and formalin were used for in vitro and in vivo tests.

Instruments used were Analytical Balance (AB 54‐S by Metter Todela, Switzerland), CBC (Human Count Plus Germany), Chiller (MDF‐U32Y; SANYO Electric Co. Ltd., Japan), Centrifugal Machine (2231 Hamburg USA), Glucometer (Infaopis Co Ltd), HPLC (Shimadzu, Japan), Incubator (MD‐MINI, Major Science USA), Ice Crusher (KT‐108; ELISA S.R. Traders), FTIR (Shimadzu, Japan), Oven (Universal Model U30; Germany), Refrigerated Centrifuge Machine (2‐16PK Sigma Laboratories Germany), Rotary Vacuum Evaporator (Heidolph Laboratories 4002 Sigma Aldrich Germany), UV Spectrophotometry (UV‐2500 Pharmape Shimadzu Japan) and Vortex Mixer (My Lab SLV‐6 Bioscience Korea).

## 3 In Silico Methodology

### 3.1 Selection and Preparation of Phytochemicals

The bioactive compounds from the n‐hexane extract of CB identified through GC–MS and HPLC analyses were selected for in silico analysis. Key compounds included Quercetin, Luteolin, Chlorogenic acid, Caffeic acid, β‐Sitosterol, and Palmitic acid. The PubChem database reclaimed the 2D structures of these phytoconstituents and transformed them into 3D structures using Open Babel. To optimize molecular geometry, energy minimization was performed using the MMFF94 force field.

### 3.2 Target Protein Selection and Preparation

Based on the literature review, analytical protein targets associated with NAFLD pathogenesis were selected. These included: peroxisome proliferator‐activated receptor alpha (PPAR‐α‐ PDB ID: 2P54), peroxisome proliferator‐activated receptor gamma (PPAR‐γ‐ PDB ID: 3DZY), AMP‐activated protein kinase (AMPK‐PDB ID: 4CFE), and sterol regulatory element‐binding protein 1c (SREBP‐1c). The targeted protein structures were prepared using AutoDock Tools by removing water molecules, co‐crystallized ligands, and heteroatoms, followed by the addition of polar hydrogens and Kollman charges. Based on the binding pocket of the native ligand, the active sites were identified.

### 3.3 Molecular Docking

Molecular docking was performed using AutoDock Vina integrated with PyRx 0.8 software. The grid box was fixed around the active sites of each protein to ensure adequate ligand accommodation. The results of docking were classified based on binding affinity (kcal/mol). Visualization of ligand‐protein interactions was conducted using Discovery Studio Visualizer and Chimera to analyze hydrogen bonding, hydrophobic interactions, and π‐π stacking.

### 3.4 ADMET and Drug‐Likeness Analysis

Using SwissADME and admetSAR tools, pharmacokinetic properties and drug‐likeness were anticipated. The parameters analyzed included Lipinski's Rule of Five, oral bioavailability, blood‐brain barrier permeability, hepatotoxicity, and mutagenicity.

### 3.5 Collection of Plant Material

The plant was collected from the local fields of Faisalabad, Pakistan. The identification of the plant needles was done by taxonomist Dr. Mansoor Hameed, Head of the Botany Department, Agriculture University, Faisalabad, Pakistan. The plant was kept in the University herbarium for future reference.

### 3.6 Extract Preparation

The whole plant was washed, dried, and crushed into powder by an electric grinder and sieved. The 1000 g of plant powder was soaked in n‐hexane for 1 week. It was shaken many times in a day. It was filtered out by using Whatman filter paper; the filtrate obtained was evaporated by using a rotary evaporator at 40°C–50°C, and 61.1 g of extract was stored in small amber jars at 4°C (Kunle et al. 2012). It was soluble in 20% Tween 80 for further use.

$$\begin{array}{cc} \text{Percentage Yield}=\; & \left(\text{Extract weight}/\text{Soaked powder weight}\right)\\ & \times 100=61.1/1000\times 100=6.2\% \end{array}$$

### 3.7 Housing of Animals

The Sprague–Dawley rats (200–300 g) were housed in individual cages on a 12‐h light/dark cycle under controlled temperature, with free access to water and diet for 28 days. All procedures were approved by the Ethical Committee of the University of Punjab, Department of Zoology, with Ref #748. The animals were divided into 6 groups, and all rats were fed the HFD. Food was prepared according to the AIN‐93 diet (Table 1). Each group contained five rats. Control conditions at room temperature (25°C ± 2°C) with a relative humidity of 45%–55% and 12‐h light and 12‐h dark cycles were maintained (Wang et al. 2016).

**TABLE 1:** Composition of HFD according to the AIN‐93 diet.

#	Nutrients	Food used in the study	HFD (g)
1	Carbohydrates	Sugar	155
2	Proteins	Albumin powder	200
3	Lipids	Animal fat 350 g and soya oil 200 g	550
4	Fibers	Ispaghul husk	50
5	Vitamin mix	Vitrum tablet	10
6	Mineral mix		35
7	Choline	Choline powder	6570

We have selected 250, 500, and 750 mg/kg doses based on previous studies demonstrating the pharmacological activity and safety of CB extracts in rodent models (Saleem, Naseer, et al. 2014; Akhtar 2021).

*Group 1*: Animals have free access to food and water *ad libitum*.

*Group 2*: Disease control group: Fatty food (60% kcal from fat) was given in the morning.

*Group 3*: Standard control group: Fatty food, along with healthy food, was administered. Silymarin 100mg/5 mL syrup was also orally provided daily in the morning for 28 days.

*Group 4*: *CB* n‐hexane extract dose of 250 mg/kg was administered with fatty food, along with healthy food orally, daily in the morning for 28 days.

*Group 5*: *CB* n‐hexane extract dose of 500 mg/kg was administered with fatty food, along with healthy food orally, daily in the morning, for 28 days.

*Group 6*: *CB* dose of 750 mg/kg was administered with fatty food along with healthy food, daily in the morning orally for 28 days.

At the end of the week, the weight of the rats was determined by using a weighing balance. On the 7th day of every week, the blood glucose level was determined by using a glucometer. On the 28th day, blood was collected through cardiac puncture, and a complete blood count (CBC) was estimated via CBC Humalyzer.

### 3.8 Biochemical Evaluation

Blood serum of rats was examined for biochemical evaluation, including alanine aminotransferase (ALT), aspartate aminotransferase (AST), alkaline phosphatase (ALKP), total bilirubin (TB), direct bilirubin (DB), LDH, triglycerides, total cholesterol, HDL‐cholesterol, LDL, VLDL, TNF‐α estimation, CRP estimation, and total antioxidant capacity.

### 3.9 Histopathological Assessment of the Liver

A portion of the liver, around 6 mm^3^, was incised in all rats. This liver part was preserved in phosphate‐buffered 10% formalin solution. A thin section of 5 μm was cut using a rotary microtome and stained with acidic base dye, i.e., hematoxylin–eosin, and slides were prepared and examined under the high‐resolution microscope at 100×. Liver histopathology was examined for steatosis, lobular inflammation, and hepatocellular ballooning using the NAFLD Activity Score (NAS). Scores were assigned as follows:

Steatosis (0–3): 0 = < 5%; 1 = 5%–33%; 2 = 34%–66%; 3 = > 66%.

Lobular Inflammation (0–3): 0 = no foci; 1 = < 2 foci per 200× field; 2 = 2–4 foci; 3 = > 4 foci.

Ballooning (0–2): 0 = none; 1 = few ballooned cells; 2 = many/prominent ballooned cells.

The total NAS score ranges from 0 to 8. Scores ≥ 5 were considered diagnostic for steatohepatitis, 3–4 as borderline, and ≤ 2 as not steatohepatitis.

### 3.10 Statistical Analysis

Statistical Analysis of the results was conducted between the NAFLD group and the Normal Control group (NCG) through Graphpad Prism 5.01.v. The analysis of results was carried out by 2‐way ANOVA followed by a Tukey's post hoc test, *p* < 0.05 considered significant.

## 4 Results

### 4.1 Molecular Docking

Different binding affinities toward NAFLD‐related targets were presented by all chosen phytochemicals. The binding energies (kcal/mol) of each compound according to protein targets are summarized in Table 2. β‐Sitosterol demonstrated the strongest binding to both PPAR‐α and PPAR‐γ, recommending potent modulation of lipid metabolism. Quercetin and Luteolin showed significant binding affinity to AMPK, a key regulator of fatty acid oxidation. Moderate binding was exhibited through all compounds for SREBP‐1c, indicating potential downregulation of lipogenesis. The docking results suggest favorable binding interactions, supporting the potential of quercetin as a therapeutic agent targeting multiple pathways in NAFLD.

**TABLE 2:** Binding affinity in kcal/mol of selected compounds with NAFLD‐related targets.

Compound	PPAR‐α (2P54)	PPAR‐γ (3DZY)	AMPK (4CFE)	SREBP‐1c (Model)
Quercetin	−8.9	−8.2	−9.3	−7.6
Luteolin	−8.7	−8.4	−9.0	−7.3
Chlorogenic acid	−7.5	−6.9	−8.7	−6.8
Caffeic acid	−7.1	−6.5	−7.8	−6.3
β‐Sitosterol	−9.4	−9.1	−8.5	−8.1
Palmitic acid	−6.2	−5.7	−6.4	−5.8

**FIGURE 1:**
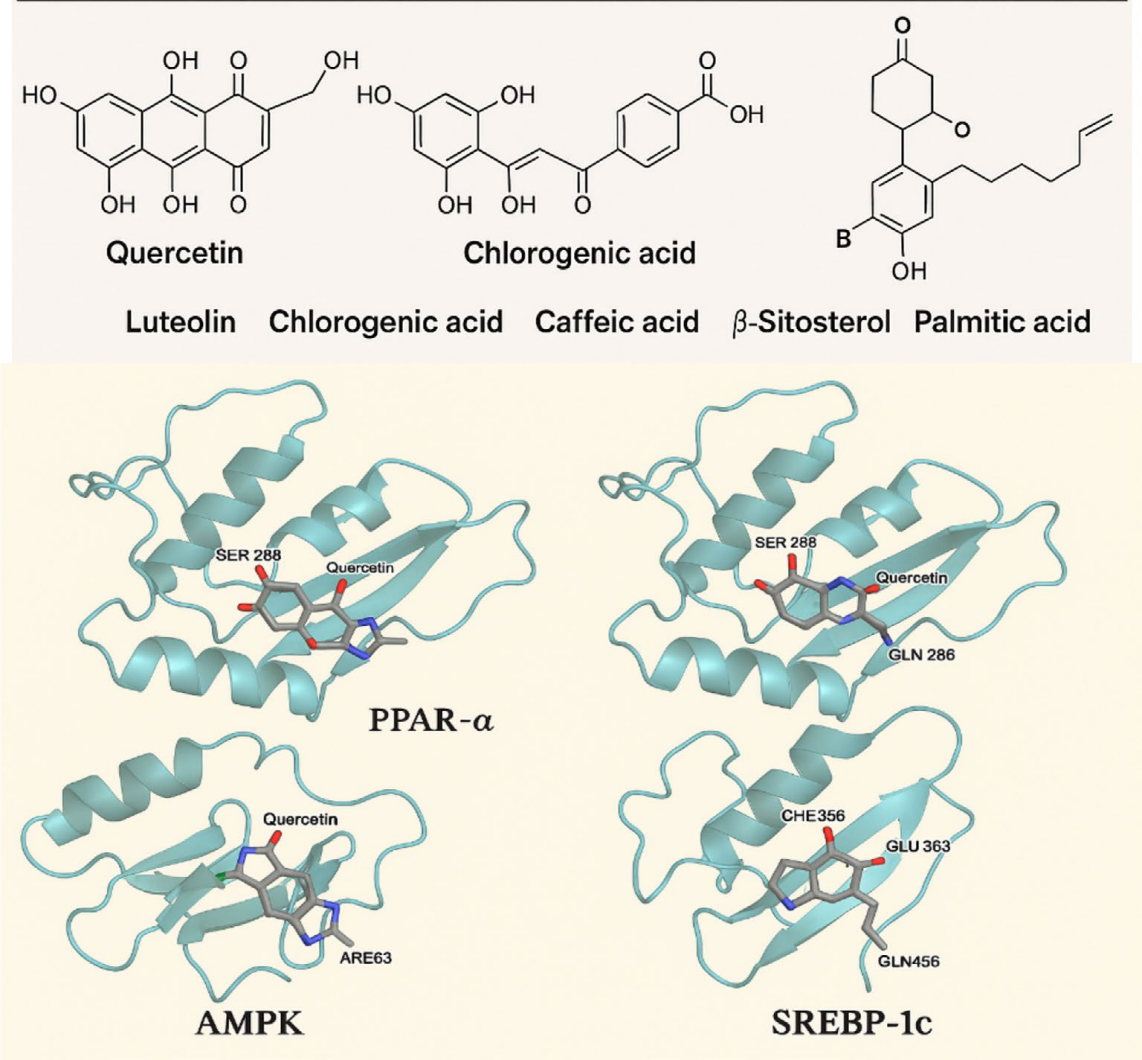
Molecular docking interactions of quercetin with four key protein targets associated with lipid metabolism and inflammation in NAFLD, PPAR‐α, PPAR‐γ, AMPK, and SREBP‐1c. Each panel shows the binding of quercetin (stick model) within the active site of the respective protein. Key interacting residues are labeled, and hydrogen bonds are indicated by green dashed lines (generated using ChatGPT).

Quercetin interacts with AMPK (4CFE). Their key interactions include hydrogen bonds with residues Asp139, Glu100, and Arg83; π‐π stacking with Tyr152, with the binding energy of −9.3 kcal/mol. This interaction has potential for AMPK activation, supporting fatty acid oxidation and anti‐steatotic effects. β‐Sitosterol mainly interacts with PPAR‐γ (3DZY). This protein–protein interaction includes hydrophobic contacts with Cys285, Leu330, and Met364, with a binding energy of −9.1 kcal/mol. It activates PPAR‐γ partial agonist activity, supporting insulin sensitization and lipid regulation (Figure 1).

### 4.2 ADMET and Drug‐Likeness Profile

All tested compounds were anticipated to be non‐mutagenic, non‐carcinogenic, and non‐hepatotoxic, promoting their safety profile. β‐Sitosterol had one Lipinski violation (molecular weight > 500) but maintained good bioavailability, as shown in Table 3.

**TABLE 3:** ADMET and drug‐likeness prediction of lead compounds.

Compound	Lipinski rule	Oral bioavailability	Hepatotoxicity	BBB penetration	Mutagenicity
Quercetin	Pass	Moderate	Low	No	No
Luteolin	Pass	Moderate	Low	No	No
Chlorogenic acid	Pass	Low	Low	No	No
β‐Sitosterol	1 Violation	High	Low	No	No

The in silico analysis suggests that phytochemicals from CB show multi‐target interactions relevant to NAFLD pathophysiology. Notably, β‐Sitosterol, Quercetin, and Luteolin demonstrated strong binding affinities toward PPAR‐α, PPAR‐γ, and AMPK, providing a molecular basis for their potential hepatoprotective and anti‐lipogenic activities. These results endorse and support the in vivo findings and suggest further advancement of CB as a powerful applicant for NAFLD management.

### 4.3 In Vivo Evaluation of CB Extracts

#### 4.3.1 Change in Body Weight

The results showed that body weight (BW) in Fatty Liver‐Induced Disease Group 2 (*p < 0.05) increased every week as compared to Standard Control Group 3.

**FIGURE 2:**
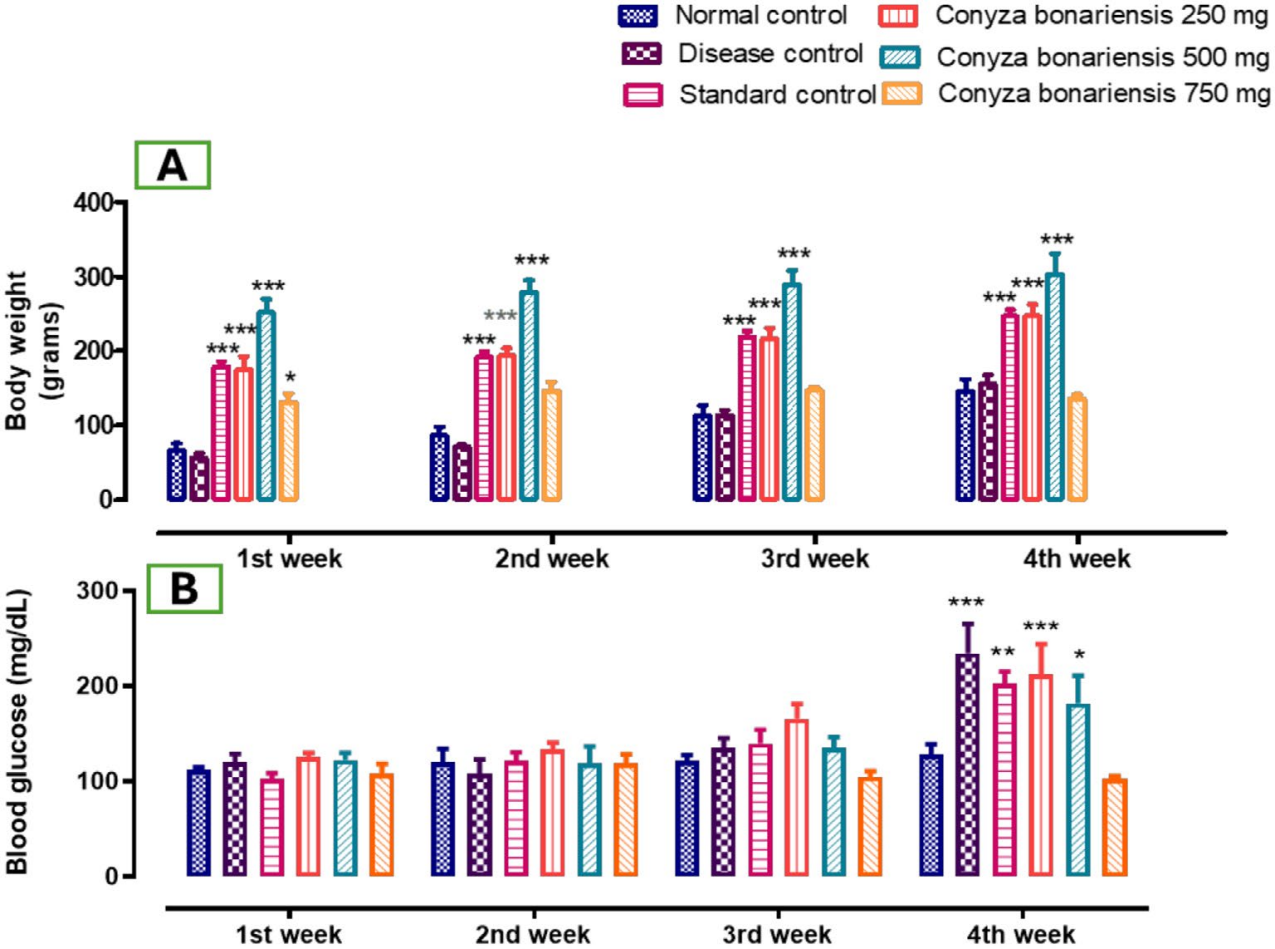
(A) Bar graph showing weekly changes in body weight over 28 days in all experimental groups (*n* = 5). Rats fed HFD exhibited a continuous increase in body weight compared to the NCG. Administration of *CB* extract (250, 500, and 750 mg/kg) resulted in a dose‐dependent attenuation of weight gain, with the highest dose showing a significant reduction relative to the disease control group. (B) Bar graph illustrating fasting blood glucose levels recorded weekly. HFD‐fed rats developed hyperglycemia, whereas treatment with *CB* extract led to significant and progressive glucose‐lowering effects. The CB 750 mg/kg group showed glucose levels approaching those of the NCG by week 4. The effects were comparable to the standard treatment group receiving silymarin (*p < 0.05, **p < 0.01, ***p < 0.001).

In treatment groups of a CB 250 mg dose, BW increased significantly in 28 days (***p < 0.01). BW on the 7th day was (186 ± 11.76), on the 14th day (199.6 ± 8.64), 21st day (226.4 ± 15.32), and 28th day (245 ± 19.70), as compared to the fatty liver‐induced group 2. Doses of CB 500 mg n‐hexane extract increased BW significantly (***p < 0.001) from the 1st week to the 4th week. BW in the 1st week was (252.2 ± 17.54), 2nd week (295 ± 12.06), 3rd week (289.4 ± 18.91), and 4th week (303.4 ± 27.64). Doses of CB 750 mg n‐hexane extract also significantly increased BW on the 7th (131 ± 11.97) and 14th day (146.6 ± 11.97 *p < 0.05). On the 21st (144.2 ± 8.26) and 28th day (136.6 ± 6.44), the BW of the 750 mg dose increased significantly (*p < 0.05). In standard group 3, BW increased significantly (**p < 0.01). On the 7th day (179.2 ± 6.94), 14th day (192.4 ± 7.11), 21st day (218.6 ± 8.09), and 28th day (233 ± 15.85) (Table 4, Figure 2A).

**TABLE 4:** Effect of *Conyza bonariensis* extract on body weight and fasting blood glucose levels in HFD‐fed rats over 28 days.

#	Groups	Body weight				Diabetes			
		1st week	2nd week	3rd week	4th week	1st week	2nd week	3rd week	4th week
1	Control	67.2 ± 9.01	87 ± 11.50	113.6 ± 13.74	146 ± 16.00	112.6 ± 2.83	120.6 ± 14.00	121.8 ± 6.011	128.2 ± 11.015
2	Disease + HFD	57 ± 5.61	71.8 ± 2.81	114 ± 6.59	156.2 ± 11.70	120.8 ± 8.41	108.6 ± 14.91	135.2 ± 10.42	234.6 ± 31.10***
3	Standard + HFD	179.2 ± 6.94***	192.4 ± 7.11***	218.6 ± 8.09***	233 ± 15.85***	103.2 ± 5.75	121.8 ± 8.99	140.2 ± 14.108	202.6 ± 12.87**
4	*CB* 250 mg + HFD	186 ± 11.76***	199.6 ± 8.64***	226.4 ± 15.32***	245 ± 19.70***	126 ± 4.08	134.2 ± 6.69	165.8 ± 15.80	212.4 ± 31.84***
5	*CB* 500 mg + HFD	252.2 ± 17.54***	295 ± 12.06***	289.4 ± 18.91***	303.4 ± 27.64***	122.6 ± 7.51	119 ± 17.98	135.6 ± 11.26	182.2 ± 29.07*
6	*CB* 750 mg + HFD	131 ± 11.97*	146.6 ± 11.97*	144.2 ± 8.26	136.6 ± 6.44	108.2 ± 10.65	119.6 ± 9.24	104.8 ± 6.13	103 ± 3.06

*Note:* Data are expressed as mean ± standard deviation (SD), *n* = 5. Statistical comparisons were made using one‐way ANOVA followed by Tukey’s post hoc test. Differences were considered statistically significant at: *p* < 0.05 (*), *p* < 0.01 (**), *p* < 0.001 (***). (*p < 0.05, **p < 0.01, ***p < 0.001 compared to disease control group).

#### 4.3.2 Change in Blood Sugar Level

Data revealed that the blood sugar level (mg/dL) in the fatty liver‐induced group 2 was (234.6 ± 31.10) on the 28th day, which was significantly (***p < 0.001) increased compared to the standard group 3. Administration of HFD along with an extract of a CB 250 mg dose, the blood sugar level in the 4th week was (212.8 ± 31.84 ***p < 0.001) significantly higher as compared to the NCG. Doses of CB 500 mg of n‐hexane extract significantly lowered blood glucose levels last week (182.2 ± 29.07), which significantly (*p < 0.05) lowered blood sugar levels, and CB 750 mg also lowered blood sugar levels (103 ± 3.06) significantly (*p < 0.05) in comparison to NCG (Table 4, Figure 2B).

### 4.4 Biochemical Analysis

#### 4.4.1 Liver Function Tests (LFTs)

**FIGURE 3:**
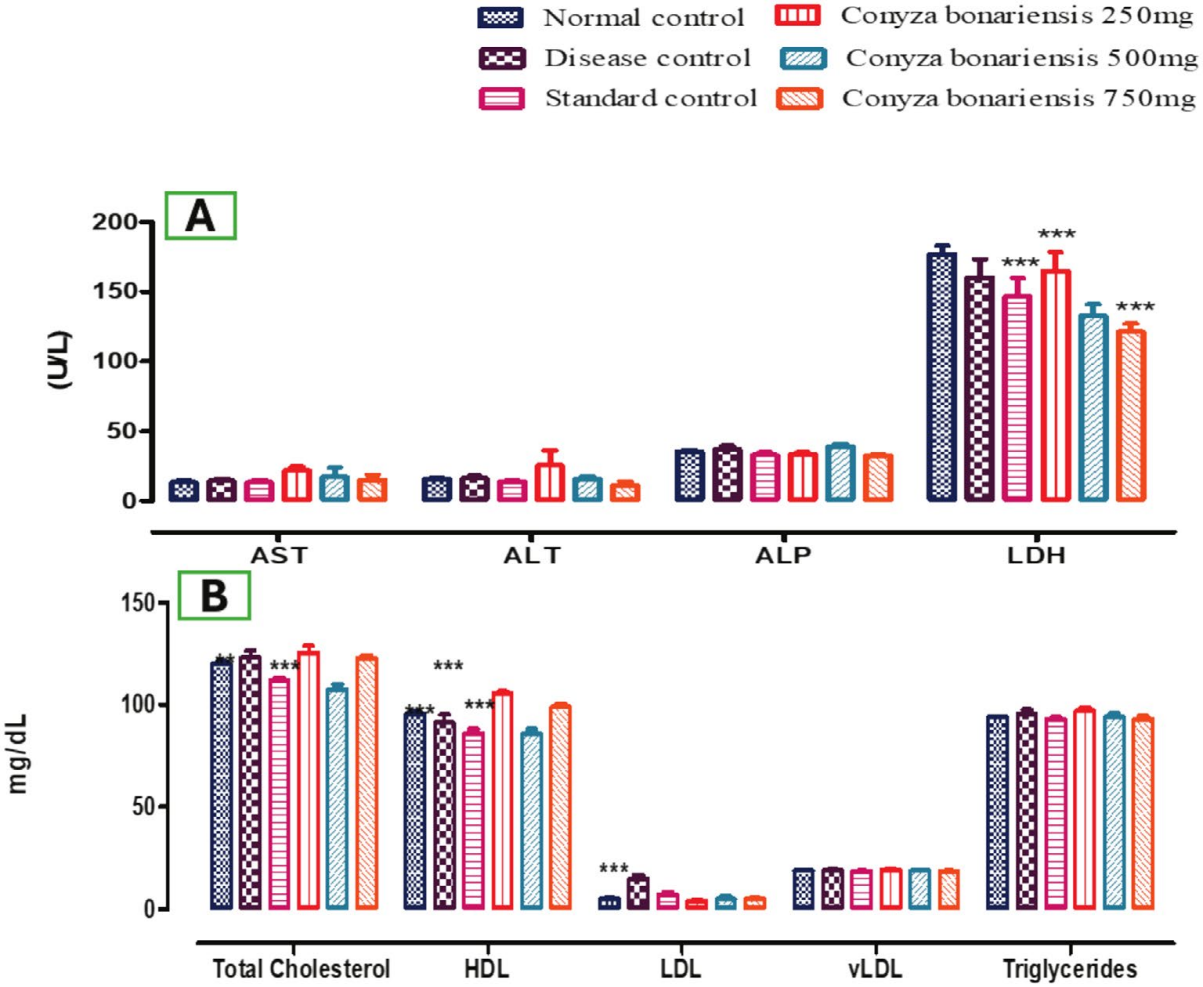
(A) Bar graph displaying liver function test (ALT, AST, ALP, bilirubin, and LDH) in all groups (*n* = 5). CB extract showed a dose‐dependent normalization of elevated hepatic enzymes. (B) Bar graph showing lipid profile markers (total cholesterol, triglycerides, HDL, LDL, VLDL) across treatment groups. Notable improvement was observed, particularly with the 500 to 750 mg CB extract doses (***p < 0.001).

The results showed that AST activity (U/L) increased significantly (*p < 0.05) in treatment groups at doses of CB 250 mg (21.99 ± 2.76), CB 500 mg (17.36 ± 6.69), CB 750 mg (15.25 ± 3.57), and the standard group (13.47 ± 1.82) as compared to the NCG (13.39 ± 1.61). Data showed that ALT activity (U/L) increased significantly in a fatty liver‐induced group (16.30 ± 1.84), while the dose of CB 250 mg (25.77 ± 10.51), CB 500 mg (15.85 ± 1.82), and CB 750 mg (11.28 ± 2.70) decreased significantly (*p < 0.05) the ALT value in comparison with the NCG (15.95 ± 1.03). ALP activity (U/L) significantly increased (*p < 0.05) in a disease control group (37.56 ± 2.16), while in different treatment groups of extract with a dose of CB 250 mg (33.68 ± 1.17), CB 750 mg (32.76 ± 1.00) and a standard group (32.98 ± 2.04) significantly decreased the ALP value (*p < 0.05) and the 500 mg (39.22 ± 1.53) dose significantly (*p < 0.05) increased the ALP as compared to the NCG (35.60 ± 1.02) as shown in Table 5 and Figure 3A.

**TABLE 5:** Effect of *Conyza bonariensis* extract on serum biochemical parameters in HFD‐induced NAFLD rats.

#	Parameters	Control	Disease control + HFD	Standard control + HFD	*CB* 250 mg + HFD	*CB* 500 mg + HFD	*CB* 750 mg + HFD
1	ALT (U/L)	15.95 ± 1.03	16.30 ± 1.84	14.16 ± 0.88	25.77 ± 10.51	15.85 ± 1.82	11.28 ± 2.70
2	AST (U/L)	13.39 ± 1.61	14.49 ± 1.64	13.47 ± 1.82	21.99 ± 2.76	17.36 ± 6.69	15.25 ± 3.57
3	ALP (U/L)	35.60 ± 1.02	37.56 ± 2.16	32.98 ± 2.04	33.68 ± 1.17	39.22 ± 1.53	32.76 ± 1.00
4	TB (mg/dL)	16.10 ± 0.05	16.44 ± 0.57	16.85 ± 0.10	16.53 ± 0.25	17.14 ± 0.15	19.66 ± 0.04
5	DB (mg/dL)	15.77 ± 0.15	16.58 ± 0.21	18.36 ± 0.28	17.82 ± 0.66	19.11 ± 0.12	26.01 ± 0.14
6	LDH (U/L)	177.18 ± 5.84	160.39 ± 12.85	146.93 ± 13.01***	165.03 ± 13.49***	133.14 ± 7.92	121.33 ± 5.78***
7	Triglycerides (mg/dLl)	93.82 ± 0.35	95.33 ± 2.18	92.91 ± 0.86	96.874 ± 1.51	93.96 ± 1.61	92.82 ± 1.50
8	HDL (mg/dLl)	95.51 ± 1.15	91.126 ± 3.92	85.86 ± 2.08***	105.54 ± 0.94***	85.79 ± 2.13***	98.82 ± 1.38
9	LDL (mg/dL)	5.09 ± 0.41	14.73 ± 1.96***	7.30 ± 1.05	3.90 ± 0.59	4.99 ± 1.29	4.94 ± 0.66
10	VLDL (mg/dLl)	18.758 ± 0.07	19.02 ± 0.43	18.58 ± 0.19	19.39 ± 0.29	18.78 ± 0.32	18.56 ± 0.30
11	TCH (mg/dL)	119.84 ± 1.39	122.92 ± 3.38	111.73 ± 1.42**	125.38 ± 3.45	107.56 ± 2.40***	122.66 ± 1.40
12	WBCs (10^9^/L)	8.579 ± 2.13***	8.82 ± 0.85	8.68 ± 1.22	11.9 ± 1.45	8.68 ± 1.63	6.64 ± 1.07
13	RBCs (10^12^/L)	4.514 ± 0.46	6.35 ± 0.10	7.60 ± 0.14	7.66 ± 0.13	6.81 ± 0.20	7.72 ± 0.15
14	Lymphocytes (10^9^/L)	4.16 ± 0.57	6.76 ± 0.69	6.28 ± 0.96	8.14 ± 1.19	6.26 ± 1.07	4.12 ± 0.56
15	Granulocytes (10^9^/L)	0.83 ± 0.10	1.34 ± 0.27	1.48 ± 0.25	2.56 ± 0.28	1.78 ± 0.37	1.32 ± 0.36
16	Hemoglobin (mg/dL)	6.82 ± 0.48	12.2 ± 0.25	13.6 ± 0.11	13.08 ± 0.24	12.1 ± 0.43	14.08 ± 0.18
17	Platelets (10^9^/L)	307 ± 88.07	462.6 ± 32.33***	540 ± 52.80***	501.8 ± 33.85***	536 ± 46.34***	524 ± 32.24***
18	TNF‐α (ng/mL)	33.8 ± 41.64	63.3 ± 5.44**	41.9 ± 4.53	55.2 ± 6.93	43.75 ± 4.68	26.22 ± 7.76
19	CRP (ng/mL)	44.02 ± 2.85	47.66 ± 6.68	57.04 ± 3.04	43.14 ± 6.5	41.18 ± 4.26	43.96 ± 3.28
20	TAOC (ng/mL)	4.12 ± 0.73	4.48 ± 0.968	3.06 ± 0.669	3.56 ± 0.552	4.8 ± 0.69	2.7 ± 0.67

*Note:* Data are expressed as mean ± standard deviation (SD), *n* = 5. Statistical comparisons were made using one‐way ANOVA followed by Tukey’s post hoc test. Differences were considered statistically significant at: *p* < 0.05 (*), *p* < 0.01 (**), *p* < 0.001 (***). (*p < 0.05, **p < 0.01, ***p < 0.001 compared to disease control group).

The results showed that bilirubin activity (mg/dL) increased significantly (*p < 0.05) in the disease rats as compared to the NCG. Simultaneous use of an HFD and various doses of n‐hexane extract at a dose of CB 250 mg (17.82 ± 0.66), CB 500 mg (19.11 ± 0.12), CB 750 mg (26.01 ± 0.14), and a standard drug (18.36 ± 0.28) (*p < 0.05) significantly increased the value of direct bilirubin activity (mg/dL) as compared to the NCG (15.77 ± 0.15). For the TB level, concomitant administration of HFD and various doses of n‐hexane extract with a dose of 250 mg (16.53 ± 0.25), 500 mg (17.14 ± 0.15) and 750 mg (19.66 ± 0.04) significantly increased (*p < 0.05) and the TB activity (mg/dl) value in the standard group (16.85 ± 0.10) decreased significantly (*p < 0.05) compared with the NCG (16.10 ± 0.05).

The results showed that LDH activity (U/L) decreased significantly (***p < 0.001) in diseased rats (160.39 ± 12.85) compared to the control group (177.18 ± 5.84). Simultaneous administration of an HFD and various doses of n‐hexane extract with a dose of CB 250 mg (165.03 ± 13.49), CB 750 mg (121.33 ± 5.78, ***p < 0.01) significantly reduced the LDH value, and a dose of CB 500 mg (133.14 ± 7.92) significantly decreased (*p < 0.05) the LDH value as compared to the NCG. The simultaneous administration of an HFD and silymarin (146.93 ± 13.01) also significantly (***p < 0.01) reduced the LDH value (U/L) as compared to the NCG shown in Table 5, Figure 3A.

#### 4.4.2 Lipid Profile

The results showed that the total cholesterol (TCH) (mg/dl) in the disease group (122.92 ± 3.38), CB extract of the 250 mg dose (125.38 ± 3.45, *p < 0.05), increased significantly. But at a CB 500 mg dose (107.56 ± 2.40, ***p < 0.001), TCH significantly decreased. The dose of CB 750 mg (122.66 ± 1.40) and the standard group (111.73 ± 1.42) also significantly (**p < 0.01) reduced the TCH (mg/dL) compared to the NCG (119.84 ± 1.39). The triglyceride level (mg/dL) in the disease group (95.33 ± 2.18), n‐hexane extract with a dose of CB 250 mg (96.874 ± 1.51) increased significantly (*p < 0.05) and a dose of CB 500 mg (93.96 ± 1.61), 750 mg (92.82 ± 1.50), and the standard group (92.91 ± 0.86) significantly (*p < 0.05) reduced the triglyceride value as compared to NCG (93.82 ± 0.35). The HDL value (mg/dL) in the disease group (91.126 ± 3.92) decreased significantly (*p < 0.05) and n‐hexane extract with a dose of CB 250 mg (105.54 ± 0.94), CB 500 mg (85.79 ± 2.13), and the standard group (85.86 ± 2.08) significantly (***p < 0.001) reduced HDL value, as shown in Table 5 and Figure 3B.

The dose of 750 mg (98.82 ± 1.38) significantly (*p < 0.05) increased the HDL value as compared to the NCG (95.51 ± 1.15). The LDL value (mg/dL) in the disease group (14.73 ± 1.96) increased significantly (***p < 0.001) and different doses of the treatment group, like CB 250 mg (3.90 ± 0.59), CB 500 mg (4.99 ± 1.29) and CB 750 mg (4.94 ± 0.66) significantly (*p < 0.05) lowered the LDL value in the standard group (7.30 ± 1.05) (*p < 0.05) increased the LDL value (mg/dL) was significantly (*p < 0.05) increased compared to the NCG (5.09 ± 0.41). The VLDL value (mg/dL) in the disease group, with different treatment doses of extract, like CB 250 mg (19.39 ± 0.29), CB 500 mg (18.78 ± 0.32), and CB 750 mg (18.56 ± 0.30), increased significantly (*p < 0.05) compared to the NCG. Simultaneous administration of the HFD and silymarin (18.58 ± 0.19) also significantly (*p < 0.05) reduced the vLDL value (mg/dL) as compared to the NCG (18.758 ± 0.07), as shown in Table 5 and Figure 3B.

#### 4.4.3 Complete Blood Count (CBC)

**FIGURE 4:**
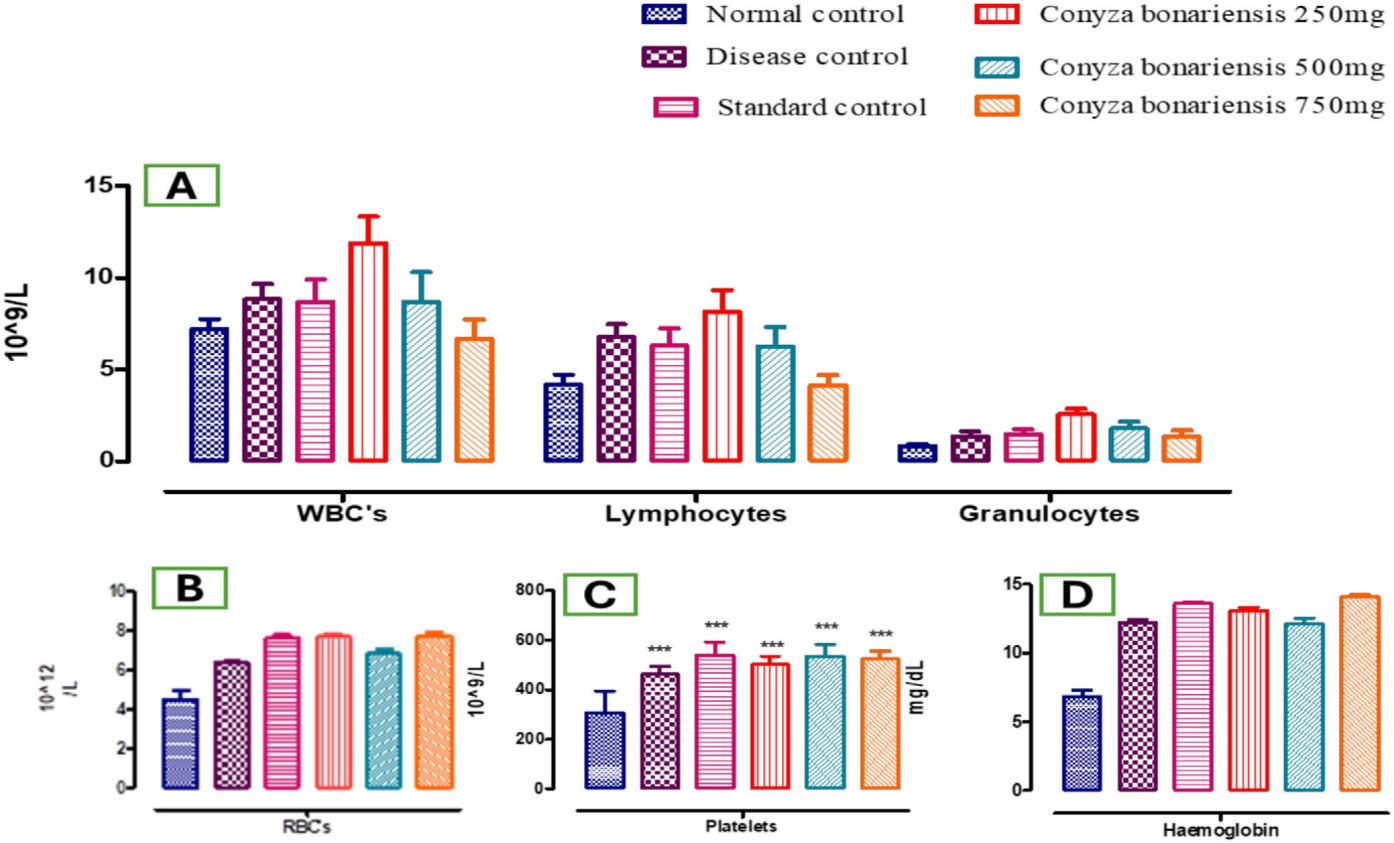
(A) Bar graph depicting white blood cell count, lymphocytes, and granulocytes. (B) Bar graph showing red blood cell count (RBC). (C) Bar graph showing platelet count in various groups. (D) Bar graph for hemoglobin levels. These hematological parameters improved significantly in CB‐treated groups, particularly at higher doses where *n* = 5 (***p < 0.001).

The results showed that the number of leukocytes (10^9^ /L) in the disease group (8.82 ± 0.85) and the standard group (8.68 ± 1.22) (*p < 0.05) was recorded. The animals administered the dose of 250 mg n‐hexane CB extract showed (11.9 ± 1.45) and 500 mg (8.68 ± 1.63), compared to the number of leukocytes significantly increased compared to the control group (85.79 ± 2.13). It was observed that the CB 750 mg dose group showed (6.64 ± 1.07, *p < 0.05) significantly decreased leukocytes in comparison with the NCG. Data revealed that the lymphocyte count (10^9^/L) in the disease group (12.2 ± 0.25), different doses of n‐hexane extract 250 mg (13.08 ± 0.24) and 500 mg (12.1 ± 0.43, *p < 0.05) significantly increased the lymphocyte count, while the dose of 750 mg (14.08 ± 0.18) (*p < 0.05) decreased while WBC count in the standard group (13.6 ± 0.11) increased significantly (**p < 0.01) as compared to NCG (6.82 ± 0.48). Data showed that the granulocyte count (10^9^/L) in the disease group (1.34 ± 0.27) and different doses of n‐hexane CB extract 250, 500, 750 mg (2.56 ± 0.28, 1.78 ± 0.37, 1.32 ± 0.36) and the standard group (1.48 ± 0.25, *p < 0.05) compared granulocyte count to the NCG (0.83 ± 0.10) as shown in Table 5 and Figure 4A.

The results showed that the number of erythrocytes (10^12^/L) in the disease group (6.35 ± 0.10), different doses of n‐hexane CB extract 250 mg (7.66 ± 0.13), 500 mg (6.81 ± 0.20), 750 mg (7.72 ± 0.15), and standard group (7.60 ± 0.14) (*p < 0.05) compared to erythrocytes in the NCG 4.514 ± 0.46 as shown in Table 5 and Figure 4B.

The results of the study demonstrated that the platelet count (10^9^/L) in the disease group (462.6 ± 32.33), the dose of 250 mg (501.8 ± 33.85), 500 mg (536 ± 46.34), 750 mg (524 ± 32.24) of extract and the standard group (540 ± 52.80) increased significantly (***p < 0.001) as compared to the NCG (307 ± 88.07) as shown in Table 5, Figure 4C.

The results showed that the hemoglobin count (mg/dL) increased significantly in the disease group (12.2 ± 0.25), various doses of CB 250 mg (13.08 ± 0.24), 500 mg (12.1 ± 0.43), and 750 mg (14.08 ± 0.18) of n‐hexane extract, and the standard group (13.6 ± 0.11) as compared to the NCG (6.82 ± 0.48) which is significant (*p < 0.05) as shown in Table 5 and Figure 4D.

#### 4.4.4 Oxidative Stress Markers

##### 4.4.4.1 Tumor Necrosis Factor‐Alpha Levels (TNF‐α)

**FIGURE 5:**
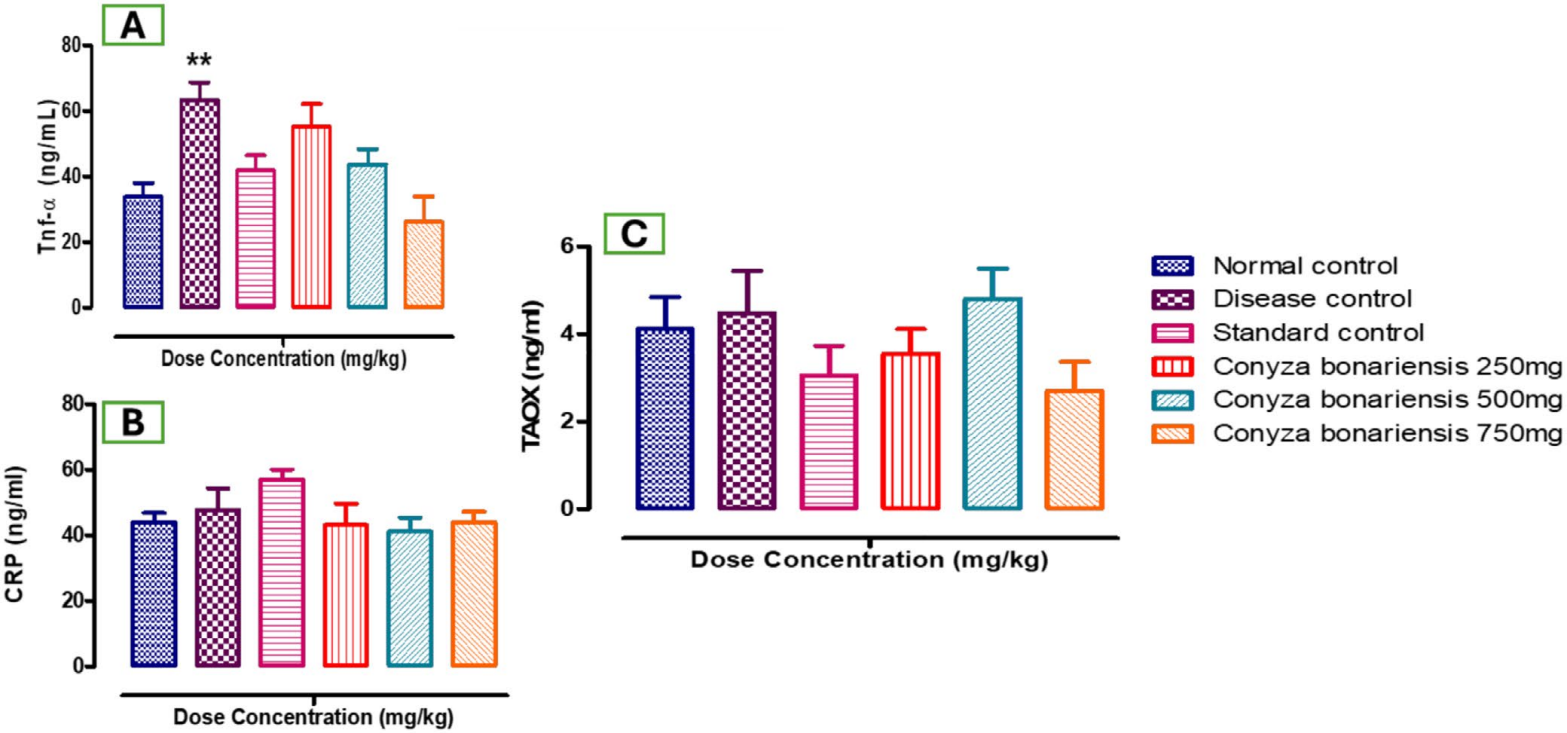
(A) Bar graph showing TNF‐α levels. HFD‐induced rats had elevated TNF‐α, which was dose‐dependently reduced by CB extract. (B) Bar graph showing C‐reactive protein (CRP) levels across all groups. (C) Bar graph presenting total antioxidant capacity (TAOC). CB extract modulated these inflammatory and oxidative stress markers significantly, where *n* = 5 (**p < 0.01).

The results showed that the TNF‐α level (ng/ml) increased significantly in the fatty liver‐induced group (63.3 ± 5.44), 250 mg (55.2 ± 6.93), and 500 mg (43.75 ± 4.68) which were significantly (*p < 0.05) increased compared to the NCG (33.8 ± 41.64) and 750 mg (26.22 ± 7.76) decreased significantly (*p < 0.05) as shown in Table 5, Figure 5A.

##### 4.4.4.2 C‐Reactive Protein Concentrations (CRP)

The results showed that the CRP level (ng/mL) increased significantly (*p < 0.05) in the fatty liver‐induced group (47.66 ± 6.68), while in the treatment group of all doses 250 mg (43.14 ± 6.5), 500 mg (41.18 ± 4.26), 750 mg (43.96 ± 3.28) as compared to the NCG (44.02 ± 2.85) which increased significantly (*p < 0.05) as shown in Table 5, Figure 5B.

##### 4.4.4.3 Total Antioxidant Capacity (TAOC)

The results showed that TAOC (ng/mL) increased significantly (*p < 0.05) in the fatty liver‐induced group (4.48 ± 0.968) in the treatment group *CB* of all doses: 250 mg (3.56 ± 0.552), 500 mg (4.8 ± 0.69), and 750 mg (2.7 ± 0.67) significantly increased (*p < 0.05) as compared to the NCG (4.12 ± 0.73). In the Standard group (3.06 ± 0.669), TAOC capacity decreased significantly (*p < 0.05) as compared to the NCG, as shown in Table 5, Figure 5C.

## 5 Histopathology

**FIGURE 6:**
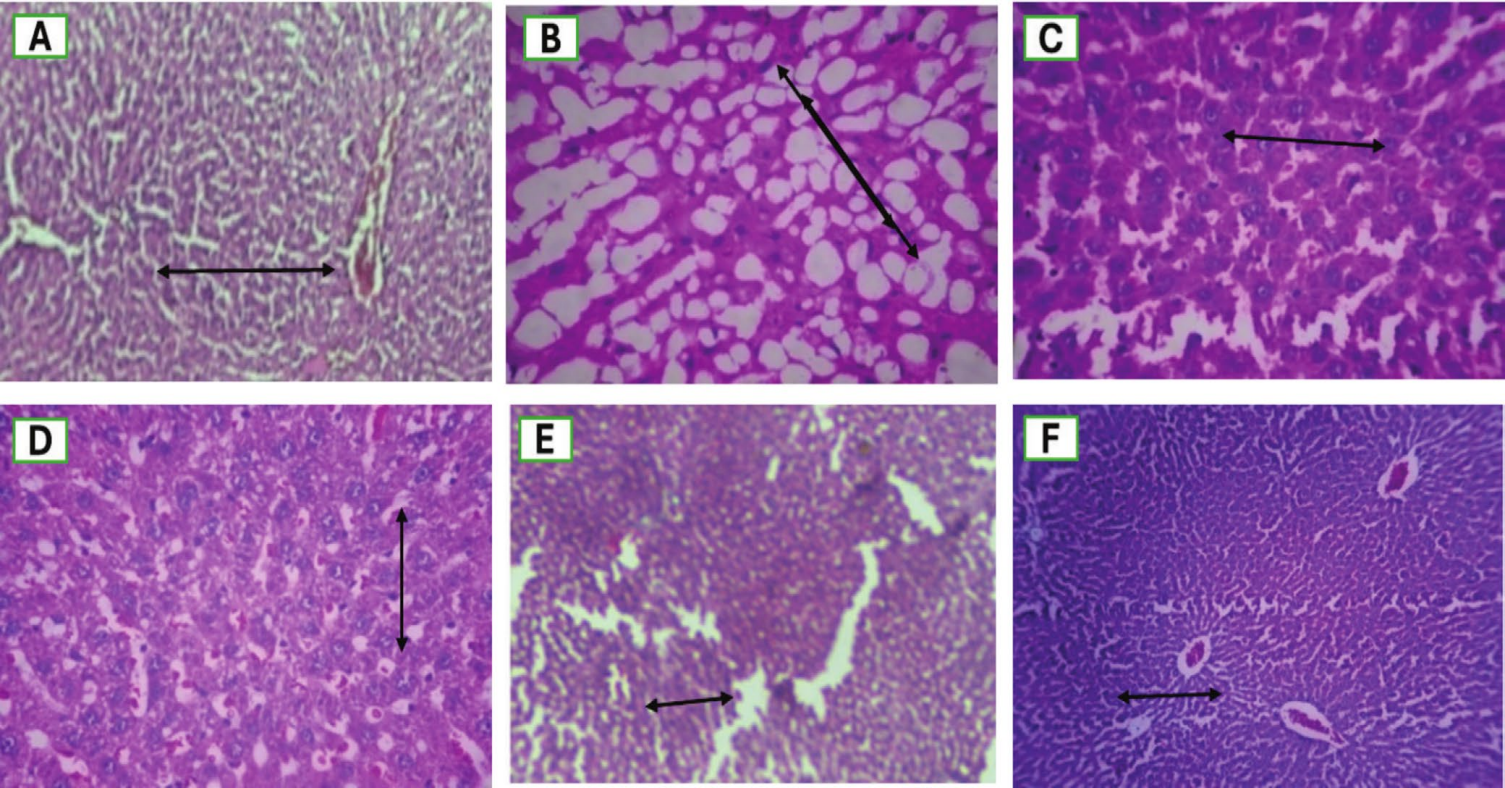
Histopathological evaluation of rat liver, where *n* = 5 at 100×. (A) NCG liver of rats shows slight fatty change with normal lobular architecture and hepatic cells with intact nucleus and nucleolus. (B) The liver of the disease control group showed markedly diffuse fatty change with microvesicular and macrovesicular changes, with excessive fat deposition and interlobular inflammation. Greater than 90% fatty change is present there. (C) In the standard control group, approximately 5% steatosis was observed in liver sections, with mild fatty changes. (D) In this group of CB 250 mg doses, perivenular mild fatty change is represented, approximately 5% steatosis is present, indicated by the red arrow. (E) In this group of CBs, 500 mg of very scant tissues were indicated, with 0% fatty change visible in this group. (F) In this group of *CB* 250 mg doses, sinusoidal and vascular congestion are present, and only 0% fatty change is there.

The following microscopic findings about steatosis and NAS score were examined in rat liver sections, as shown in Figure 6 and Table 6.

**TABLE 6:** NAS score of histopathological images of liver sections stained with H&E from experimental groups (×100 magnification).

Group	Steatosis	Ballooning	Inflammation	Total NAS Score
A—Normal Control	0	0	0	0
B—HFD (NAFLD model)	3	2	3	8
C—Silymarin 100 mg/kg	1	0	1	2
D—CB 250 mg/kg	2	1	2	5
E—CB 500 mg/kg	1	1	1	3
F—CB 750 mg/kg	1	0	1	2

## 6 Discussion

The current study was used to evaluate and determine the hepatoprotective effects of *CB* against HFD‐induced NAFLD (Wang et al. 2016). In NAFLD, for 4 weeks, rats gained weight constantly in different doses of *CB*, 250, 500, and 750 mg, compared to the NCG, in which silymarin was administered. Weight gain is the basic factor responsible for NAFLD (Saleem, Naseer, et al. 2015).

Hyperglycemia would be considered when the fasting blood glucose level is 110 mg/dL, and the random blood glucose level is 140 mg/dL. In animal groups treated with the extract of *CB*, 250, 500, and 750 mg were 169.2, 152.4, and 105.6 mg/dL, respectively. With the increase in the dose of the extract, the blood glucose level decreases (Farzaneh and Carvalho 2015; Noreen et al. 2018). Transfer of free fatty acids to the liver is due to high blood glucose levels and low insulin production, which in turn leads to steatosis and vice versa (Alam et al. 2016). The hemoglobin level in the *CB* 500 mg group had the lowest level of Hb among the different dosing groups. Hemoglobin acts as an oxygen and carbon dioxide carrier in the body (Naseer et al. 2023). Elevated hemoglobin levels associated with NAFLD serve as a protective agent and antioxidant effect in the normal range. The functional group of hemoglobin is heme, which causes an inflammatory reaction when oxidized, resulting in lipid peroxidation and oxidative stress. With the increase in the severity of the disease, the level of hemoglobin increases (Eapen et al. 2018).

Current research demonstrated that CBC values showed that the RBC count was significantly lower in the disease control group compared to the standard control group. However, the *CB* doses of 250, 500, and 750 mg groups showed low RBC count compared to the NCG. RBCs had significant importance in the transport of oxygen and nitrogen in the body and carbon dioxide to the lungs, and the scavenging of free radical oxygen species (Saleem, Qadir, et al. 2014). The liver is responsible for the chemical detoxification of compounds. It degrades RBCs through macrophages, and it has a major antioxidant function (Munkong et al. 2023). When RBCs fail to scavenge free radical oxygen species, they cause ROS and iron release from RBCs, increase oxidative stress, and worsen fatty liver. Increased levels of RBCs are present in fatty liver disease due to decreased capacity of liver detoxification, which further worsens the condition. RBC levels were high in NAFLD as compared to normal liver conditions (Niu et al. 2024).

WBC count was significantly high in the CB 500 mg group among all dosing groups as compared to the NCG. WBC count was a prominent marker of inflammation. Lymphocytes and neutrophils are closely linked to the occurrence of NAFLD. Lymphocytes controlled the synthesis of inflammatory mediators from macrophages (Zhang et al. 2019). The platelet level was significantly higher in *CB* 500 mg compared to NCG, and the lymphocyte level decreased with an increase in the dose of *CB* (Saleem, Abbas, et al. 2015). Different biochemical parameters like lipid profile, LFTs, RFTs, and albumin test vary with increased hemoglobin levels. Hem group is an actual serum marker of early NAFLD diagnosis (de Back et al. 2014). Thrombocytopenia is a prominent indicator of NAFLD. It is a cheap method for the identification of the severity of NAFLD. Platelet count decreases in the last stages of cirrhosis, as compared to the disease progression stage (Mouskeftara et al. 2024).

LFTs are the markers that describe the severity of liver damage. AST is a liver enzyme that plays an important part in the diagnosis of NAFLD and serves as an indicator of liver injury. High levels of ALT are related to symptoms of metabolic syndrome (Wang et al. 2017). Obesity is strongly related to high levels of ALT. HFD is strongly linked with elevated levels of ALP (Saleem, Abbas, et al. 2014). ALP is a biomarker of steatohepatitis in patients with hepatic fibrosis. ALT is a basic biomarker of hepatic damage that describes the intensity of fibrosis (Juárez‐Hernández et al. 2018). It has a direct correlation with triglyceride levels, blood glucose levels, and an inverse correlation with hemoglobin and HDL. ALT level shows different levels in cirrhosis as compared to the initial stages of steatosis (Tian et al. 2016). ALT level was minimal in the *CB* 750 mg group as compared to NCG. Similarly, the AST level was the lowest in *CB* 750 mg among every treatment group, in contrast to the NCG. AST and ALKP are independent biomarkers of steatohepatitis, and in the case of liver fibrosis, it has twice the value as compared to normal values (Panke et al. 2020). High total and direct bilirubin levels play an important role in reducing the NAFLD threat. In hepatic cell carcinoma, low bilirubin levels indicate liver inefficiency and low bilirubin production (Anjum et al. 2025).

High levels of bilirubin decrease oxidative stress and have anti‐inflammatory effects, which decrease inflammatory mediators like interleukin‐6 and interleukin‐1. LDH is a biochemical enzyme that converts pyruvate to lactate under anaerobic conditions (Hadizadeh et al. 2017). In hepatic cell carcinoma, LDH is released from cancer cells. High serum levels of LDH are associated with hypoxia in tumor cells. Its value was lowest in comparison to the NCG in the treatment group of the 750 mg dose. Hypertriglyceridemia is an independent parameter in NAFLD (Faloppi et al. 2016). High lipid levels in the blood are also associated with most patients with NAFLD. The 750 mg *dose* group has the lowest triglyceride level. LDL level is high, and hypo‐HDL level is present. LDL level was lowest at 250 mg among other groups. The normal value of HDL is present in the 250 mg group. But at 750 mg, it has the lowest vLDL level. In steatohepatitis, insulin could not stop the synthesis of glucose and vLDL (Tomizawa et al. 2014). Cholesterol metabolism and production are maintained by the liver. But in the case of NAFLD, cholesterol does not metabolize and accumulates in the liver, which in turn increases cholesterol levels in the blood. Abnormality in normal cholesterol metabolism progresses toward the severity of NAFLD and cardiovascular disease (Paquissi 2016).

C‐reactive protein is directly related to NAFLD because CRP is produced by the liver and adipose tissue, and this biomarker is significantly higher in NASH compared to simple steatosis. CRP in CB dose 500 mg has a low CRP level in comparison with NCG, while the standard control group has the highest level of CRP in comparison with the NCG. Abnormal fat deposition in the liver and obesity lead to pro‐inflammatory mediators such as TNF‐α, IL‐6, and IL‐8, and reduce the synthesis of anti‐inflammatory factors such as IL‐10 and adiponectin (Anjum et al. 2024; Stojsavljević et al. 2014). For example, in NAFLD patients, the levels of inflammatory biomarkers TNF‐α, IL‐6, and IL‐8 increase. TNF‐α has a significant part in the development of the disease by activating various inflammatory mechanisms. As a result, the HDL level drops, and cholesterol accumulation in the body increases (Sumida et al. 2013).

TNF‐α is a prominent biochemical marker that describes the severity of NAFLD. Its level is higher in steatohepatitis as compared to the control and the normal healthy group (Chalasani et al. 2018). The *CB* 750 mg dose‐treated group has the lowest TNF‐α levels as compared to the control group. Improvement in insulin insensitivity, an increase in HDL, and a decrease in triglyceride levels indicate low TNF‐α levels in the body. TAOX capacity indicates the level and product of lipid peroxidation, which is higher in NAFLD as compared to NCG (Papadopoulos et al. 2023). Oxidative stress in liver steatosis leads to the progression of the disease with the production of inflammation markers and variations in the metabolic pathways. Progressive stages of NAFLD lead to an increased level of ROS, free radicals, and a low level of SOD, catalase, and other antioxidant enzymes (Balakrishnan et al. 2021).

The study of histopathology of the liver in different animal groups demonstrated a degree of steatosis and fat deposition. HFD‐induced fatty liver depicts a clear deposition of fats and steatosis with microvascular and macrovascular variations (Zhang et al. 2024). This is because of the release of inflammatory mediators, the accumulation of cholesterol in hepatocytes, and insulin insensitivity. While the silymarin‐administered group had almost 5% fat deposition, extracts of CB showed < 5% fatty changes in the liver (Lu et al. 2022). Various histological changes appear in the liver biopsy. > 5% fat deposition in hepatocytes with pathological changes like ballooning of hepatocytes, inflammation, and hepatocellular injury with fibrosis is termed steatohepatitis, while < 5% fat deposition without any hepatocellular injury and pathological changes is termed steatosis (Ali et al. 2025). There are differences between histopathological changes in pediatrics and adults suffering from NAFLD. On a histopathological basis, the release of inflammatory mediators and fatty change in extracts of 750 mg showed more efficacy compared to the standard control group and 250 mg and 500 mg dose groups.

## 7 Conclusion

The present study demonstrates that CB n‐hexane extract, particularly at a dose of 750 mg/kg, exhibits significant hepatoprotective, anti‐inflammatory, and metabolic regulatory effects in high‐fat diet‐induced NAFLD in rats. The extract improved liver function markers, lipid profiles, glucose levels, and oxidative stress indicators, while histopathological analysis confirmed reduced hepatic steatosis and inflammation. In silico docking, strong interactions of key phytochemicals, especially quercetin, luteolin, and β‐sitosterol, with NAFLD‐related targets (PPAR‐α, PPAR‐γ, AMPK, and SREBP‐1c), support the mechanistic basis for its efficacy. These findings suggest that CB holds promising potential as a natural therapeutic agent for the prevention and management of NAFLD. However, further clinical studies are warranted to validate its safety and effectiveness in humans.

## Author Contributions

**Bassam S. M. Al Kazman, Sadia Rana, and Taha Muhammad:** conceptualization, writing the original draft, in silico and in vitro analysis, mechanistic study, methodology, and review of the manuscript. **Faiza Naseer, Ethar Abdullah Mudhish, and Mohammed A. Alshamrani:** in vitro analysis, methodology, writing, and proofreading of the article and review of the manuscript. **Omaish S. Alqahtani, Mater H. Mahnashi, and Mohammad Saleem:** methodology; investigation; supervision, funding resources; data curation.

## Disclosure

The authors reviewed and edited all AI‐assisted content to ensure accuracy and scientific integrity.

## Consent

The authors have nothing to report.

## Conflicts of Interest

The authors declare no conflicts of interest.

## Data Availability

All data generated or analyzed during this study are included in this published article.
